# Interpretable machine learning reveals daytime and nighttime forest fire point drivers in Guizhou

**DOI:** 10.1016/j.isci.2026.116799

**Published:** 2026-07-15

**Authors:** Yunlin Zhang, Zhiyang Li, Long Chen

**Affiliations:** 1 School of Biology Sciences, Guizhou Education University, Guiyang 550018, China; 2 Key Laboratory of Ecology and Management on Forest Fire in Universities of Guizhou Province, Guiyang 550018, China; 3 Shenzhen Zhongtian Jingcheng Environmental Protection Consulting Co., Ltd, Shenzhen 518000, China

**Keywords:** MODIS active fire points, daytime and nighttime, fire risk grade zoning, Ensemble model, SHAP analysis, Guizhou Province

## Abstract

Daytime and nighttime forest fire risk reflects contrasting atmospheric, fuel, and human activity conditions, with implications for time specific fire management. We analyzed moderate resolution imaging spectroradiometer (MODIS) active fire points in Guizhou Province from 2009 to 2024 and integrated meteorological, topographic, vegetation, anthropogenic, and socioeconomic variables to model all, daytime, and nighttime fire points occurrence. Random Forest (RF), eXtreme Gradient Boosting (XGBoost), Light Gradient Boosting Machine (LightGBM), and ensemble models were compared, and SHAP analysis was used to interpret driver contributions and generate relative fire risk grade zoning maps. Fire points showed strong spring concentration and clustering in southern and southwestern Guizhou. Daytime high-risk areas were broader and more continuous, whereas nighttime high-risk areas were concentrated in fewer core zones. Humidity, temperature, rainfall, wind, and normalized difference vegetation index (NDVI) were shared drivers, while human related factors differed between daytime and nighttime models. This study provides evidence for time-specific forest fire monitoring, risk assessment, and prevention in mountainous regions by revealing daytime and nighttime differences in fire points and their drivers.

## Highlights

• Forest fire points peaked in spring, with 81.2% occurring from February to April
• High-risk areas are primarily concentrated in the southern and southwestern parts of Guizhou
• Meteorological factors and NDVI dominated daytime and nighttime fire-point drivers
• Ensemble model AUCs were 0.9687, 0.9637, and 0.9701 for all, daytime, and nighttime data

## Graphical abstract

**Figure:**
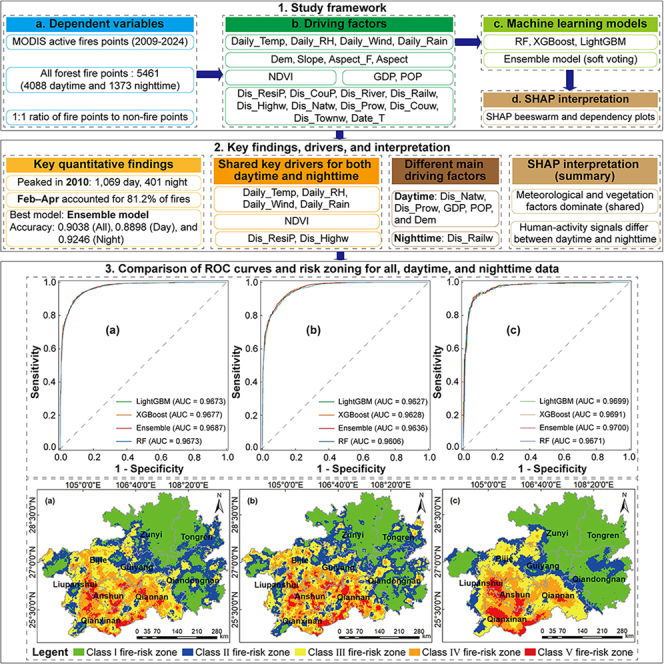

## Teaser

Artificial intelligence; artificial intelligence applications; environmental monitoring

## Introduction

Forest fires are a key disturbance factor that affects global climate change and forest ecosystem succession, directly influencing seed germination, community distribution, biodiversity, and ecosystem services, while also posing a severe threat to human life, property, and social stability.^1^^,^^2^ In the context of global climate change, the frequency of forest fires is becoming an increasingly severe issue. According to predictions from the United Nations Environment Programme, extreme forest fire events are expected to increase by 14%, 30%, and 50% by 2030, 2050, and the end of this century, respectively.^3^ In the face of the growing forest fire risk, systematically revealing the spatiotemporal dynamics and driving mechanisms of forest fire under the influence of climate change is a critical prerequisite for improving the precision and effectiveness of forest fire prevention and control.

The classic theory of forest fires suggests that the decrease in nighttime temperatures and the increase in humidity lead to reduced evaporation and higher moisture content in surface fuels, thus forming a “nighttime barrier” that inhibits the occurrence and spread of forest fires. As a result, the frequency and severity of forest fires at night are typically significantly lower than during the day.^4^^,^^5^ However, recent asymmetric climate warming is altering this pattern.^6^ Studies have shown that, under global climate change, the nighttime warming rate significantly exceeds that of the daytime, which has had a considerable impact on the diurnal dynamics of forest fires. For example, between 2003 and 2020, global nighttime fire intensity increased by 7.2%. This change has led to a weakening of the traditional “nighttime barrier” effect, presenting new challenges for forest fire prevention and suppression.^5^ Therefore, exploring the spatiotemporal patterns of nighttime fires and revealing the mechanisms of forest fire occurrence under diurnal and nocturnal variations is of significant theoretical and practical importance for forest fire risk management in the context of climate change.

The occurrence of forest fires (both day and night) is jointly controlled by various environmental and anthropogenic factors. The spatiotemporal distribution patterns and driving mechanisms of forest fires remain relatively stable over a certain period.^7^^,^^8^ Environmental factors primarily include meteorological conditions, vegetation indices, and topographic features. Among these, meteorological conditions play a critical role; for example, a decrease in relative humidity significantly increases fire risk.^9^ Topographic factors, such as elevation and slope, influence fire risk by altering local hydrological and thermal conditions and the moisture content of fuels. For instance, an increase in slope can reduce the soil’s water retention capacity, accelerate the drying of fuels, and thus increase the likelihood of forest fires.^10^^,^^11^ Anthropogenic factors mainly affect the probability of fire sources, such as the accessibility of forests, the intensity of socioeconomic activities, and eco-tourism during holidays, all of which can alter the spatial and temporal distribution of fire sources.^12^ The relationships between these factors and forest fire occurrence are complex, often exhibiting nonlinear responses and potential threshold effects.^13^^,^^14^ Machine learning methods, due to their ability to handle complex interactions and nonlinear relationships between factors, have been widely applied in fire risk prediction and the analysis of driving mechanisms.^15^^,^^16^

However, existing studies on forest fire prediction and driving factor have largely treated all fire detections or overall fire risk as the analytical target. Relatively few studies have predicted and compared daytime and nighttime forest fire detections within a unified framework of data sources, explanatory variables, and modeling procedures. Previous research has shown that the natural fire-suppressing caused by lower nighttime temperature and higher humidity is being weakened due to climate warming, leading to increasing nighttime fire activity and persistence.^4^^,^^17^ These studies highlight the importance of changing nighttime fire risk, but they have mainly focused on temporal trends in nighttime fire activity and their climatic context. Systematic understanding remains limited as to whether nighttime forest fire detections differ from daytime detections in terms of predictive characteristics, dominant drivers, and spatially susceptible areas. Because daytime and nighttime fires differ in meteorological conditions, fuel-moisture dynamics, and rhythms of human activity, modeling them jointly may obscure the variable contributions and nonlinear responses specific to nighttime fire occurrence. Therefore, developing separate models for daytime and nighttime forest fire predictions within a unified analytical framework, and comparing their driving mechanisms, provides an important basis for deepening our understanding of nighttime fire risk and for advancing temporally differentiated fire prevention and management strategies.

Guizhou Province, located in the typical karst mountainous region of southwestern China, is characterized by abundant forest resources, terrain has significant undulations, intersecting agricultural and forestry areas, and complex, variable meteorological conditions terrain has significant undulations.^18^ It is a high-risk area for forest fires and also a representative region for conducting differential analysis of forest fire points between daytime and nighttime. Studies have shown that forest fire activity in this region is significantly influenced by the El Niño-Southern Oscillation,^19^^,^^20^ and under the context of climate change, a clear trend of increasing nighttime fire risk may emerge. By the end of 2024, forest cover in Guizhou had reached 63.3%, and its extensive forest resources provide a suitable basis for forest fire detection monitoring and driving-factor analysis. Meanwhile, forest fires in Guizhou are mainly caused by human factors, with human factors accounting for over 96% of recorded events. Human disturbances, including agricultural burning, ritual burning, residential production and daily activities, and traffic-related activities, exhibit distinct diurnal rhythms,^21^ which may lead to differences in ignition exposure and spatial distribution between daytime and nighttime forest fire detections. In addition, the complex karst terrain can influence local hydrothermal conditions, fuel drying processes, and nighttime firefighting safety, while steep slopes, rugged mountains, and limited road accessibility further increase the difficulty of detecting, responding to, and suppressing nighttime fires. The superposition of a strong anthropogenic ignition background and challenging mountainous firefighting conditions suggests that daytime and nighttime forest fire detections in Guizhou may differ in their driving structures and spatial distribution patterns, highlighting the regional management relevance of nighttime fire prediction and driving-factor analysis.

Therefore, using the DAY_NIGHT attribute in the moderate resolution imaging spectroradiometer (MODIS) MCD14ML active fire data, this study developed forest fire occurrence prediction models for all, daytime, and nighttime fires within a unified framework of forestland spatial constraints, selected driving factors, and machine-learning modeling. Shapley additive explanations (SHAP) analysis was further used to compare the contribution strength, effect direction, and nonlinear response patterns of the dominant drivers under different temporal scenarios. The specific objectives were to (1) characterize the temporal variations and spatial distribution differences between daytime and nighttime forest fires in Guizhou; (2) identify and compare the key drivers and response characteristics associated with the relative occurrence propensity of daytime and nighttime forest fires; and (3) evaluate the predictive performance of different machine-learning models across temporal scenarios and generate forest fire risk level zoning maps. This study will systematically clarify the mechanisms of forest fire occurrence under diurnal and nocturnal variations in Guizhou, providing scientific evidence for regional forest fire management and contributing to the theoretical understanding of nighttime fires, which is of significant value in reducing fire-related hazards.

## Results

### Spatial and temporal distribution characteristics of fires

**Figure 1:**
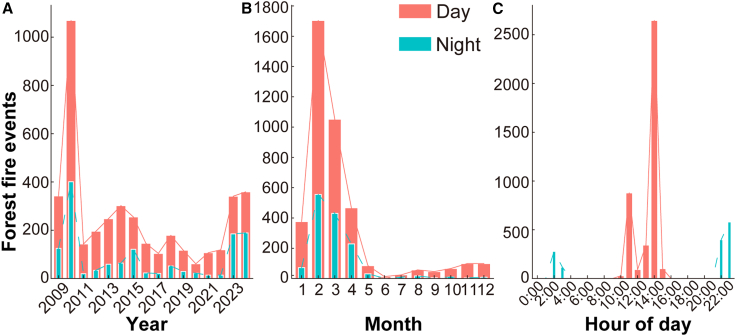
Cumulative number of daytime and nighttime fire points across multiple time scales (A) Across the year, (B) month, and (C) hour.

Figure 1 shows the temporal distribution characteristics of cumulative MODIS-detected forest fire points in Guizhou from 2009 to 2024. During the study period, the cumulative number of daytime fire points was markedly higher than that of nighttime fire points, with a ratio of approximately 3:1. The interannual variation trends of daytime and nighttime fire points were generally consistent (Figure 1A), both reaching their peaks in 2010, with 1,069 daytime fire points and 401 nighttime fire points. The monthly cumulative results showed that forest fire points were mainly concentrated from February to April, accounting for 81.2% of the total fire points (Figure 1B). February had the highest number of fire points, with 2,259 records, accounting for 41.4% of the total, and the monthly peaks of both daytime and nighttime fire points occurred in this month. At the hourly scale (Figure 1C), daytime fire were mainly concentrated between 10:00 and 15:00, with at 14:00 accounting for 64.7% of all daytime fire points. Nighttime fire was mainly concentrated during 22:00–23:00 and 02:00–03:00, accounting for 71.2% and 28.7% of all nighttime fire points, respectively.

**Figure 2:**
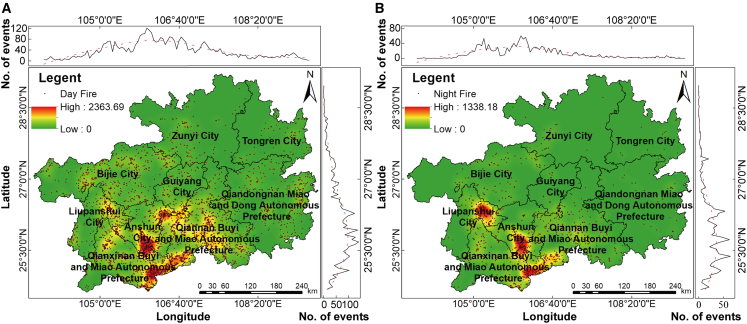
Spatial kernel density map of forest fire points in Guizhou from 2009 to 2024 (A) Daytime kernel density distribution map and (B) is nighttime. The color gradients from light to dark representing the spatial density of fire points from low to high. The red dashed line represents the fitted curve.

Figure 2 illustrates the differences in daytime-nighttime spatial kernel density distributions of forest fire points in Guizhou from 2009 to 2024. Overall, both daytime and nighttime forest fire points exhibited a clear spatial aggregation pattern, with high-density areas mainly distributed in southern and southwestern Guizhou, whereas most areas in the northern and eastern parts of the province showed relatively low fire point densities. The high-density areas of daytime fire points covered a wider spatial extent and formed a relatively continuous high-value distribution from southern to southwestern Guizhou (Figure 2A). In contrast, nighttime fire points had a lower overall density. Their high-density areas were still mainly located in southern and southwestern Guizhou, but their spatial extent was relatively contracted, showing a more concentrated patch-like pattern (Figure 2B).

### Correlation of driving factors and multicollinearity diagnosis

Table 1 presents the results of the Spearman rank correlation analysis and variance inflation factor (VIF)/generalized VIF (GVIF) multicollinearity diagnosis for the three training datasets. The results showed that no pair of continuous variables in any of the three training sets reached the threshold for high correlation. In the all-fire and daytime training sets, the variable pair with the highest correlation was Dis_Highw and Dis_Railw, with Spearman correlation coefficients of 0.658 and 0.658, respectively. In the nighttime training set, the highest correlation was observed between Daily_RH and Daily_Rain, with a correlation coefficient of 0.681. All of these correlation coefficients were below the preset threshold, indicating that no strong correlation existed among the continuous driving factors.

**Table 1:** Results of Spearman rank correlation analysis and multicollinearity diagnosis

Dataset	Sample size	Most strongly correlated variable	|r_s_|	Maximum VIF	Maximum equivalent value
All	7,646	Dis_Highw and Dis_Railw	0.658	2.357	1.010
Daytime	5,724	Dis_Highw and Dis_Railw	0.658	2.424	1.013
Nighttime	1,924	Daily_RH and Daily_Rain	0.681	2.704	1.027

Further multicollinearity diagnosis showed that the maximum VIF values in the all-fire, daytime, and nighttime training sets were 2.357, 2.424, and 2.704, respectively, all of which were well below the threshold of 10. The equivalent values for the multi-category variable Date_T were 1.010, 1.013, and 1.027, respectively, also indicating no obvious multicollinearity. Therefore, no severe correlation or multicollinearity problems were found among the selected driving factors, and all variables were retained for subsequent analysis.

### Identification of main drivers of forest fires

Significant variables were identified based on permutation importance analysis combined with statistical significance testing (*p* < 0.05) (Table S1). For the daytime period, 12 key driving factors were retained (Table S2), including meteorological factors (Daily_Temp, Daily_RH, Daily_Rain, Daily_Wind), topographic factor (Dem), vegetation factor (NDVI), anthropogenic factors (Dis_ResiP, Dis_Highw, Dis_Natw, Dis_Prow), and socioeconomic factors (population density [POP], gross domestic product [GDP]). For the nighttime period, eight factors were retained (Table S3). These were broadly consistent with the daytime set in terms of meteorological and vegetation factors, but differed in anthropogenic factors: Dis_Railw replaced Dis_Natw and Dis_Prow as a significant predictor, while POP, GDP, and Dem were not selected. Using the all dataset, 10 factors were identified (Table S4), integrating shared characteristics of both daytime and nighttime conditions. This set included the core meteorological and vegetation factors, retained key anthropogenic variables (Dis_ResiP, Dis_Highw, Dis_Natw), and also preserved GDP and Dem as factors with persistent influence.

### Model prediction results

**Figure 3:**
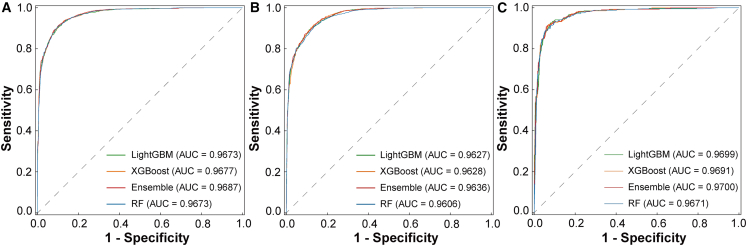
Comparison of ROC curves among different models under different temporal scenarios (A) All-fire dataset; (B) daytime dataset; and (C) nighttime dataset.

Table 2 and Figure 3 show that all four models exhibited strong discriminatory ability across the three datasets, with AUC values exceeding 0.96. For the all-fire dataset, its accuracy, F1-score, and AUC were 0.904, 0.904, and 0.969, respectively. For the daytime, the corresponding values were 0.890, 0.889, and 0.964, while for the nighttime they were 0.925, 0.925, and 0.970, respectively. In comparison, the daytime was slightly lower overall than that for the all-fire and nighttime datasets, whereas the nighttime achieved the highest overall discriminatory performance. Although the performance improvement of the ensemble model was limited, it maintained a relatively stable overall advantage across three datasets and was therefore selected for subsequent model interpretation and forest fire point risk zoning analysis.

**Table 2:** Evaluation metrics of different models across different time periods

Type	Model	Accuracy	Sensitivity	Specificity	Precision	Recall	F1	AUC
All	RF	0.9032	0.9035	0.9029	0.9030	0.9035	0.9033	0.9673
	XGBoost	0.9011	0.9042	0.8980	0.8987	0.9042	0.9014	0.9677
	LightGBM	0.9008	0.8993	0.9023	0.9020	0.8993	0.9006	0.9673
	Ensemble	0.9038	0.9048	0.9029	0.9031	0.9048	0.9039	0.9687
Day	RF	0.8825	0.8866	0.8785	0.8794	0.8866	0.8830	0.9606
	XGBoost	0.8875	0.8874	0.8850	0.8911	0.8874	0.8882	0.9628
	LightGBM	0.8891	0.8899	0.8883	0.8884	0.8899	0.8892	0.9627
	Ensemble	0.8898	0.8907	0.8915	0.8856	0.8907	0.8893	0.9637
Night	RF	0.9148	0.9197	0.9100	0.9108	0.9197	0.9153	0.9672
	XGBoost	0.9234	0.9319	0.9173	0.9185	0.9319	0.9242	0.9692
	LightGBM	0.9197	0.9270	0.9124	0.9137	0.9270	0.9203	0.9700
	Ensemble	0.9246	0.9343	0.9124	0.9143	0.9343	0.9251	0.9701

### Explanation of model decision mechanism

**Figure 4:**
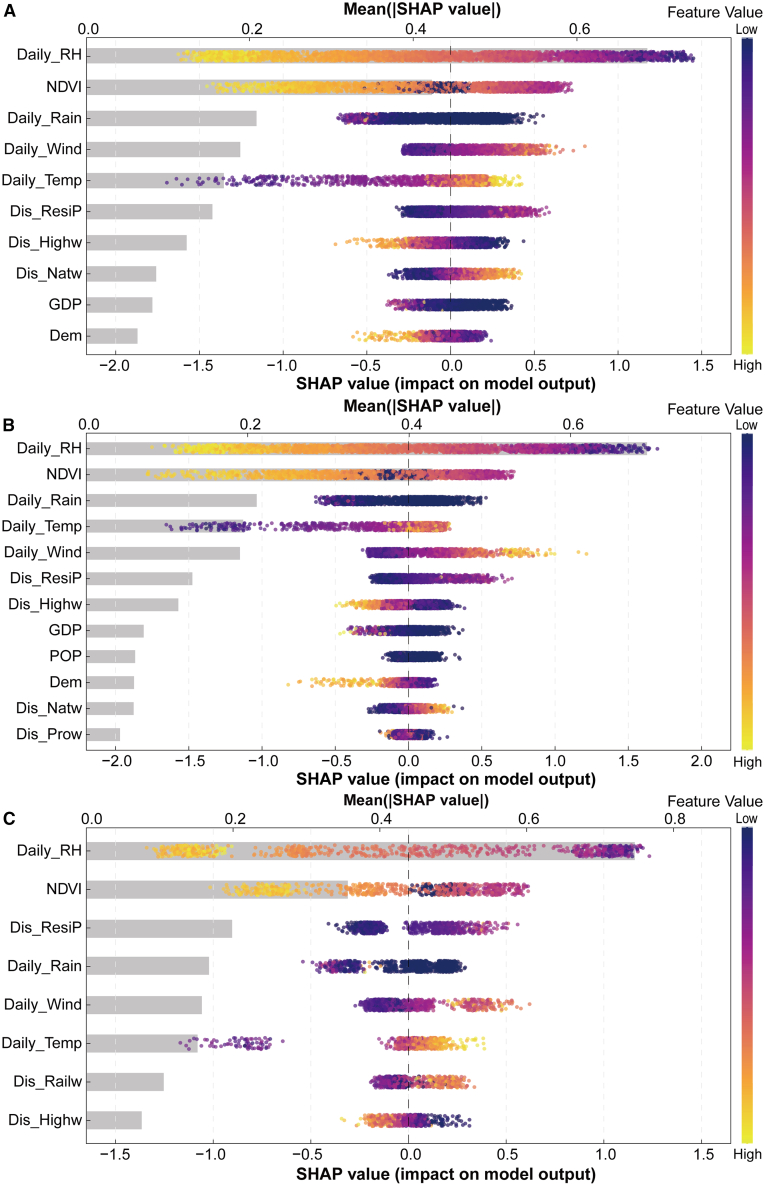
SHAP-based interpretability analysis of the forest fire prediction model (A) All-fire dataset; (B) daytime dataset; and (C) nighttime dataset. Gray bars denote mean absolute SHAP values, indicating overall variable importance, while colored points show sample-level SHAP values and corresponding feature values. The *x* axis indicates the direction and magnitude of each variable’s contribution, and the purple-to-yellow gradient represents increasing feature values.

Figure 4 shows that the main explanatory variables in the all-fire, daytime, and nighttime models were dominated by meteorological and vegetation factors. Daily_RH had the highest mean absolute SHAP value in three datasets, making it the primary variable influencing model predictions. NDVI consistently ranked among the most important variables, while Daily_Rain, Daily_Wind, and Daily_Temp also made relatively large contributions. The SHAP beeswarm plots showed that higher Daily_RH and Daily_Rain were generally associated with negative SHAP values, whereas higher Daily_Temp and Daily_Wind mostly showed positive contributions, indicating that dry, warm, and windy conditions increased the relative occurrence propensity of forest fire points. Compared with the stable contributions of meteorological and vegetation factors, anthropogenic and topography related variables showed certain differences across scenarios. In the all-fire and daytime models, variables such as road distance, GDP, POP, and digital elevation model (DEM) were among the main explanatory factors. In the nighttime model, the contribution of Dis_Railw was more prominent.

**Figure 5:**
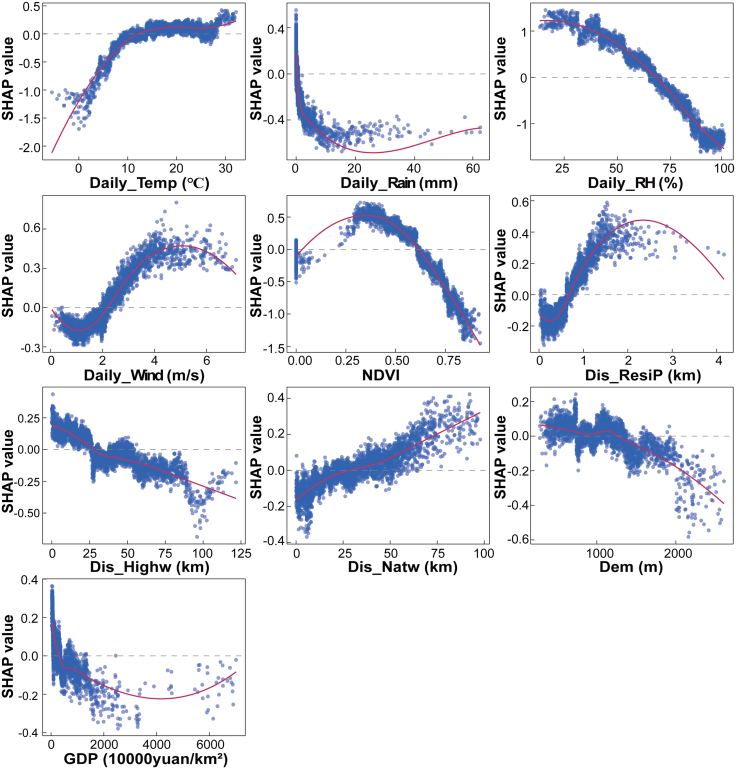
SHAP dependency plots of fire risk driving factors in the all-fire dataset The blue points represent individual fire point samples, and the red solid line represents the fitted curve. The same applies below.

**Figure 6:**
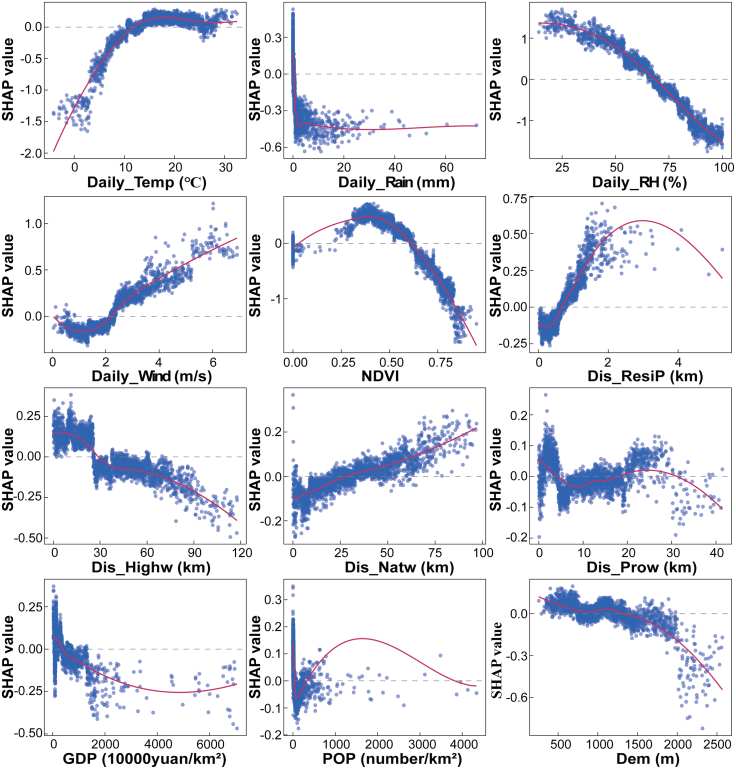
SHAP dependency plots of fire risk driving factors in the daytime dataset

**Figure 7:**
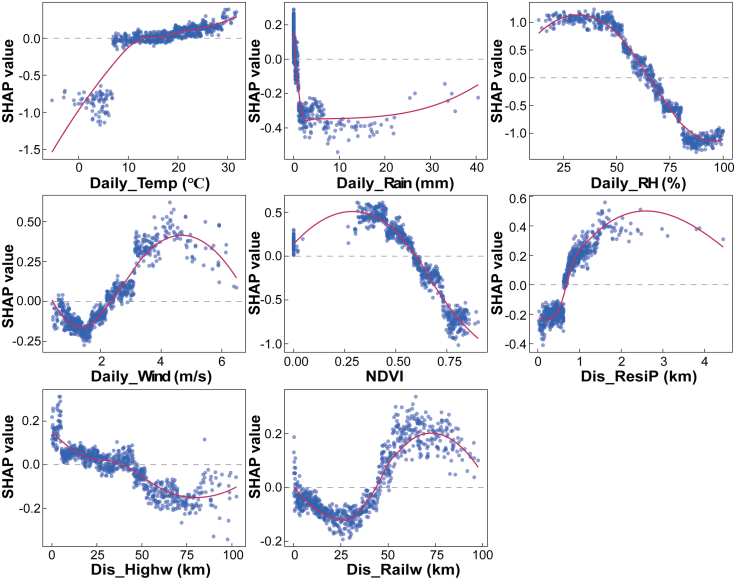
SHAP dependency plots of fire risk driving factors in the nighttime dataset

The SHAP dependence plots showed that the main driving factors in the three-datasets models exhibited clear nonlinear response patterns (Figures 5, 6, and 7). Across the three datasets, meteorological and vegetation factors showed generally consistent response directions. Daily_RH exhibited a continuous negative trend with increasing values, with SHAP values decreasing markedly under high-humidity conditions. Daily_Rain was generally dominated by negative contributions. Daily_Temp gradually shifted from a negative contribution to a weak positive contribution as temperature increased and tended to stabilize at higher temperature ranges. Daily_Wind shifted from a negative contribution at low wind speeds to a positive contribution under moderate to high wind speeds. NDVI showed a distinct unimodal response in all three models, with SHAP values increasing within the low-to-moderate value range but decreasing rapidly and becoming negative under higher NDVI conditions.

The response characteristics of anthropogenic and socioeconomic factors differed between daytime and nighttime scenarios. Dis_ResiP showed a nonlinear relationship of first increasing and then decreasing in the three datasets models, indicating that its contribution was relatively higher at intermediate distances. In the all-fire and daytime models, Dis_Highw generally showed a negative trend with increasing distance, whereas Dis_Natw showed an overall positive trend. The daytime model also showed certain response patterns for Dis_Prow, POP, GDP, and DEM. Among them, DEM generally decreased with increasing elevation, while POP exhibited a trend of first increasing and then decreasing. In contrast, accessibility variables in the nighttime model were mainly represented by Dis_Highw and Dis_Railw. Dis_Railw showed a relatively clear nonlinear response, but it did not follow a simple monotonic pattern in which shorter distance corresponded to higher SHAP contributions.

Overall, the response characteristics of meteorological and vegetation factors were relatively stable across the three models, whereas the daytime model showed richer responses involving socioeconomic, topographic, and multiple road-accessibility variables. The nighttime model, by contrast, more strongly reflected the roles of meteorological and vegetation factors, as well as specific accessibility variables such as distance to railways. These findings indicate that modeling daytime and nighttime forest fire points separately helps identify time-specific responses that are difficult to fully capture in an overall model.

### Division of forest fire risk zones

**Figure 8:**
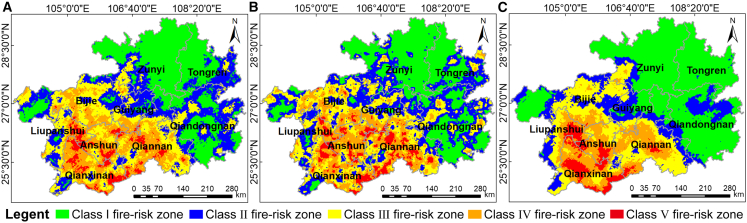
Forest fire risk zoning in Guizhou (A) all-fire dataset; (B) daytime dataset; and (C) nighttime dataset. All maps were classified into five risk grades.

Based on the probability predictions of the ensemble model, a forest fire risk zoning map was generated for Guizhou (Figure 8). The results revealed marked spatial heterogeneity in forest fire risk, with evident regional clustering. High-risk and relatively high-risk areas are primarily concentrated in the southern and southwestern parts of Guizhou, covering regions such as Qiandongnan, Qiannan, Qianxinan, and Anshun. Medium-risk and lower-risk areas are mainly located in the central and northeastern parts of the province, including parts of Guiyang, Tongren, Zunyi, and Bijie. Compared to daytime fires, the high-risk areas for nighttime fires are smaller, with clearer boundaries, and the low-risk areas are more continuous. Overall, the forest fire risk in Guizhou follows a “high in the south, low in the north; high in the west, low in the east” spatial pattern.

## Discussion

### Spatiotemporal characteristics of daytime and nighttime forest fire points

This study revealed the spatiotemporal differentiation of daytime and nighttime forest fire points in Guizhou from the perspectives of interannual, seasonal, satellite overpass detection period, and spatial distribution. At the interannual scale, the number of fire points in 2010 was markedly higher than in other years, which may be related to the persistent drought in the preceding period and the accumulation of understory litter and damaged vegetation following the freezing disturbance in 2008.^22^ At the seasonal scale, both daytime and nighttime forest fire points were mainly concentrated from February to April, indicating a clear spring concentration of forest fire points in Guizhou. During this period, the study area is usually characterized by increased wind speed and relatively low humidity,^23^ which can reduce the moisture content of understory fuels. Meanwhile, outdoor fire-use activities associated with spring farming and ritual burning are relatively concentrated. The combined effects of increased fuel availability and expanded anthropogenic ignition exposure windows lead to a marked increase in the number of forest fire points. After May, with increasing precipitation, vegetation greening, and reduced fire-use activities, the numbers of both daytime and nighttime fire points declined substantially. In terms of satellite overpass detection periods, daytime fire points were mainly concentrated between 10:00 and 15:00, with relatively high values around 14:00. This period usually corresponds to higher air temperature, lower relative humidity, and stronger near-surface drying conditions, which are more favorable for active burning to be detected by satellites.^24^^,^^25^ Nighttime fire points were mainly concentrated in two periods, 22:00–23:00 and 02:00–03:00. It should be noted that MCD14ML records active fire points detected during Terra and Aqua satellite overpasses, rather than the actual ignition time of fires. Therefore, the bimodal distribution of nighttime fire points reflects the detection characteristics of active burning pixels within the MODIS nighttime observation windows and cannot be directly attributed to peaks in ignition activity during specific nighttime periods.^17^ Nevertheless, fire points that can still be detected under the generally lower temperature and higher humidity conditions at night indicate that some fires may remain actively burning during nighttime, underscoring the practical importance of nighttime forest fire monitoring.

At the spatial scale, the high-density areas of both daytime and nighttime forest fire points were mainly concentrated in southern and southwestern Guizhou, and the locations of the two types of fire points were generally similar. This suggests that these areas may be persistently influenced by the combined effects of terrain, vegetation fuel conditions, and human activities. Compared with nighttime fire points, the high-density and sub-high-density areas of daytime fire points covered a wider spatial extent, which may be related to more active human production, daily activities, transportation, and forest-edge activities during the daytime.^26^ The spatial extent of nighttime fire points was relatively reduced, but they remained concentrated in core areas similar to those of daytime fire points, indicating that nighttime fire occurrence may have stronger spatial selectivity. These spatial differences further suggest that forest fire risk assessment in Guizhou should not only focus on long-term stable high-incidence areas but also consider daytime–nighttime differences in subsequent driving-factor interpretation and time-specific management.

### Comparison of model performance with previous studies

In this study, the ensemble model achieved relatively better overall predictive performance in the three datasets. However, its improvement over the best single model was limited, with an increase of 0.0006–0.0012 in accuracy and only 0.0001–0.0010 in AUC. This may be because Random Forest (RF), eXtreme Gradient Boosting (XGBoost), and Light Gradient Boosting Machine (LightGBM) are all tree-based ensemble learning methods, and because they used the same training samples and driving factors, their prediction and error characteristics were somewhat similar, thereby limiting the additional gain provided by simple soft-voting ensemble learning.^27^ Therefore, the advantage of the ensemble model should be understood as a limited improvement in predictive performance and an enhancement of result robustness, rather than a substantial increase in accuracy compared with individual models. In addition, the nighttime model generally achieved higher accuracy and AUC than the daytime model, suggesting that satellite-detected nighttime fire points may occur under more concentrated environmental constraints and are therefore easier to distinguish. By contrast, daytime fire points may be influenced by more diverse human activities and environmental conditions, increasing model discrimination difficulty.

Mishra et al. predicted forest fire susceptibility in Odisha, India, using MODIS fire points and 19 influencing factors, and reported a validation accuracy of more than 94% for the RF model.^28^ Sarkar et al. integrated climatic, topographic, biophysical, and anthropogenic factors to model forest fire susceptibility in northeastern India, with the RF model achieving the highest AUC value of 0.87.^29^ Guria et al. predicted forest fire occurrence probability in the Similipal Biosphere Reserve, India, using Sentinel-2 data and multiple machine-learning models, and the optimal GBM model achieved an AUC of 0.8461.^30^ These studies suggest that tree-based models and their ensembles can effectively capture complex nonlinear relationships between forest fire points and multi-source environmental factors. However, model performance cannot be strictly compared across studies because of differences in study area, sample construction, data resolution, influencing factors, and validation methods. Unlike previous studies, this study constructed separate models for daytime and nighttime scenarios within a unified analytical framework. Its main contribution lies in revealing the daytime-nighttime differences in driving mechanisms that may be obscured by an overall model.

### Analysis of differences in driving factors between daytime and nighttime fires

SHAP analysis further revealed the main drivers of the relative occurrence propensity of forest fire points in Guizhou and their daytime-nighttime differences. Overall, meteorological and NDVI made large contributions across the three datasets, indicating that forest fire points were primarily constrained by both fuel dryness and the vegetation fuel background. Among these factors, as Daily_RH increased, its SHAP values showed an overall continuous decline, indicating that higher air humidity may reduce the likelihood of active fire occurrence by increasing fine-fuel moisture content. This is consistent with previous forest fire danger studies showing the suppressive effect of relative humidity on fire occurrence.^31^ Daily_Temp generally made a positive contribution at higher value ranges, indicating that increasing temperature may enhance evaporation and promote fuel drying, thereby increasing the relative occurrence propensity of fire points.^32^ Its contribution tended to level off at high temperatures, suggesting that when fuels are already extremely dry, air temperature is no longer a limiting factor for forest fire occurrence. Daily_Rain showed an overall negative effect, whereas the contribution of Daily_Wind increased at higher value ranges, reflecting the role of rainfall in maintaining fuel moisture and the role of wind speed in promoting fuel drying and sustaining combustion conditions, respectively.^33^^,^^34^ The effect of wind speed was more consistent in daytime fires, whereas its effect on nighttime fires showed greater dispersion, which may be related to lower near-surface wind speeds and higher spatial heterogeneity in forests at night.

NDVI showed a unimodal nonlinear response in three-datasets, with SHAP contributions first increasing and then decreasing. When NDVI was low, limited vegetation cover and insufficient fuel continuity resulted in a lower fire point propensity. As NDVI increased, fuel load and continuity increased, providing a more favorable fuel basis for fire point occurrence.^35^ However, when NDVI increased further, canopy shading and a moister surface environment associated with dense vegetation reduced flammability, leading to a decline in fire point propensity.^36^ This unimodal response indicates that forest fire point occurrence in Guizhou is jointly constrained by fuel amount, fuel continuity, and fuel moisture status.

In contrast to the relative consistency of meteorological and vegetation factors, anthropogenic and socioeconomic variables showed more pronounced differences between daytime and nighttime scenarios. Daytime fire points were more strongly affected by residential proximity, road accessibility, and population–economic activity, suggesting that daytime production, transportation, and forest-edge fire use may increase potential ignition-source exposure. The SHAP response of distance to residential points was nonlinear: very short distances may be associated with limited fuel conditions due to higher built-up land proportions and more timely fire-source management; forest-edge transition zones around residential areas showed higher fire point propensity because fuels and human disturbance coexist; and with further increasing distance, anthropogenic ignition exposure weakened and the contribution declined.^37^ In the nighttime scenario, Dis_Railw showed a more prominent explanatory contribution, indicating that linear transport corridors and their surrounding areas may be associated with the distribution of nighttime datasets. Although previous studies have found that distance to railways is an important factor influencing the spatial distribution of human-caused forest fires,^38^ this study was based on remotely sensed fire points and distance variables and therefore could not directly identify the specific ignition source of individual fire points. The related causal interpretation still needs to be verified using field-based fire-cause investigations or finer-scale human activity data. Overall, road and socioeconomic variables made more evident contributions during the daytime, whereas distance to railways was more prominent at night, suggesting that daytime and nighttime datasets may be influenced by different types of anthropogenic activity backgrounds. This further highlights the need to model and interpret daytime and nighttime fire points separately.

In addition, Dem generally showed a negative contribution in the all-fire and daytime analytical scenarios, which may be related to lower temperature, higher humidity, weaker human activity intensity, and reduced accessibility in high-elevation areas.^39^ Overall, the SHAP analysis not only identified stable cross-period drivers such as relative humidity, temperature, rainfall, wind speed, and NDVI, but also revealed daytime-nighttime differences in human activity and spatial accessibility factors. Compared with assessing forest fire point risk based only on an overall model, scenario-specific interpretation for daytime and nighttime fire points can more clearly identify the main risk sources under different temporal contexts and provide a basis for developing time-specific prevention and control measures.

### Potential changes in forest fire risk under nighttime warming

Lower nighttime air temperature and higher humidity generally help increase the moisture content of fine fuels, reduce combustion intensity, and provide a relatively favorable window for fire suppression. However, under climate warming, nighttime warming and atmospheric drying may weaken this natural fire-suppressing effect. Balch et al. reported that nighttime fire-weather conditions are intensifying globally and that the suppressive effect of nighttime conditions on fire activity is weakening.^4^ Freeborn et al. further found that the persistence of nighttime fire activity has increased markedly under drier conditions and in larger fire events.^40^ This implies that future changes in forest fire risk may not only involve intensified high fire-danger conditions during the daytime but also include an expansion of nighttime fire activity periods and an increased likelihood of sustained burning at night.

This study found that although nighttime forest fire points in Guizhou were fewer than daytime fire points, they still showed clear spatial clustering and interpretable environmental response patterns. In particular, meteorological factors such as humidity and temperature played important roles in explaining the relative occurrence propensity of nighttime fire points. These findings indicate that nighttime fire points are not a negligible low-risk phenomenon in the complex mountainous forest environment of Guizhou. If nighttime warming and drying conditions continue to intensify in the future, some areas that were previously constrained by humid nighttime environments may become more capable of sustaining active burning, thereby increasing pressure on nighttime monitoring, continued suppression, and firefighter safety management. Because this study did not conduct future climate scenario simulations, the previous discussion is intended to clarify the risk implications of our findings under nighttime warming, rather than to quantitatively predict future changes in nighttime forest fire points in Guizhou. Future climate variability and extreme weather events may further affect nighttime fire occurrence in Guizhou. Droughts, heatwaves, and prolonged rain-free periods could reduce fuel moisture and weaken nighttime humidity recovery, increasing the likelihood of sustained burning after sunset.^4^^,^^5^

### Daytime-nighttime fire risk zoning and forest fire management

The high-risk areas identified in this study were concentrated in southern and southwestern Guizhou, generally consistent with the spatial kernel density map of fire points, indicating that these areas require sustained attention. However, the forest fire point risk zones derived from the optimal model reflect spatial differences under model-predicted conditions, rather than forest fire danger grades based on actual fire occurrence probabilities. Similarly, previous studies have used remote sensing indicators and machine-learning models to map forest fire probability and support the identification of priority prevention areas and management decision-making.^41^ The daytime and nighttime zoning results further revealed differences in their distribution patterns. High-risk grades for daytime fires covered a larger area and formed more continuous patches, consistent with the spatial “outward expansion” of forest fire risk under daytime conditions. During the daytime, production and daily activities, travel, agricultural burning, and forest-edge activities are more active, making ignition sources more likely to occur in transition zones around core areas. In contrast, areas with high relative susceptibility at night were more spatially contracted and mainly concentrated in a few core areas. This spatial “convergence” may indicate that, under relatively lower nighttime temperature and higher humidity, fine-fuel moisture content is less likely to reach the ignition threshold, making sustained fire difficult to develop even when ignition sources are present.^25^ The zoning based on all data integrated the daytime outward expansion and nighttime convergence mechanisms, resulting in a spatial pattern between the two. This result is more suitable as a long-term average risk background map, whereas daytime-nighttime differentiated zoning is more appropriate for time-specific control and differentiated deployment.

Forest fire management authorities in Guizhou should strengthen coordinated control of key areas and key periods. Areas in southern and southwestern Guizhou that show high risk in both daytime and nighttime scenarios should be prioritized for patrols, fire-source inspections, and monitoring equipment deployment during the spring fire-prevention period. Daytime prevention should focus on areas with strong human activity, such as residential surroundings, road-accessible areas, and agricultural-forest transition zones, with strengthened field fire-use patrols, public awareness and guidance, and dynamic monitoring during key periods. Although nighttime fire risk is relatively lower, continuous monitoring, residual-fire and rekindling inspections, and emergency duty should still be strengthened in core susceptible areas, with particular attention to nighttime patrols and fire-source inspections around transport infrastructure, especially railways, and surrounding forestlands. It should be emphasized that distance to railways reflects a spatial association background and cannot be used to directly determine the specific source of ignition. Overall, daytime-nighttime differentiated zoning can provide management authorities with more targeted spatial references than a single overall zoning map, thereby supporting forest fire prevention resource allocation and patrol arrangements by region and time period.

### Limitations of the study

This study systematically analyzed the daytime-nighttime differentiation of forest fire occurrence in Guizhou and explored the driving mechanisms using multiple models, but several limitations remain. First, the MODIS fire point data have a spatial resolution of 1 km and record active fires detected during satellite overpasses. They may therefore miss small, short-duration, or cloud- and fog-affected fires,^42^ and cannot directly represent the actual ignition time of forest fires. Second, the daily-scale meteorological factors, monthly NDVI, and socioeconomic factors used in this study can reflect the overall environmental background, but are insufficient to characterize fine-scale factors, such as local temperature and humidity, fuel moisture content, and land surface temperature at the exact time of satellite overpass.^43^ In addition, model performance was evaluated using stratified random partitioning, but further analyses of spatial autocorrelation among fire points, uncertainty, and confidence intervals for prediction results were not conducted. Therefore, the current model performance mainly reflects discriminatory ability under the internal sample conditions of the Guizhou study area, while its spatial generalizability, prediction stability, and cross-regional applicability require further examination. Future studies could incorporate spatial autocorrelation analysis, repeated sampling, and uncertainty analysis to more rigorously evaluate model robustness. In addition, near-real-time meteorological data, fuel moisture content, land surface temperature, and other variables that more directly reflect daytime and nighttime fuel conditions could be introduced, while LiDAR or unmanned aerial vehicle data could be used to characterize the spatial structure of fuels. Methodologically, while maintaining model interpretability, future studies could compare the applicability of deep-learning or spatiotemporal modeling frameworks and conduct external validation using fire point data from independent years or other typical mountainous forest regions, thereby improving the reliability of daytime and nighttime forest fire point prediction and risk identification. However, our method is specifically designed for Guizhou; for other regions, different methods may be more appropriate, and the transferability of our approach to other areas requires further validation.

## Resource availability

### Lead contact

Requests for further information and resources should be directed to and will be fulfilled by the lead contact, Yunlin Zhang (zhangyunlin@gznc.edu.cn).

### Materials availability

This study did not generate new materials.

### Data and code availability

• Data: all data acquisition URLs are listed in the article.
• Code: all original code is available on Github (https://github.com/3078599139/code.git).
• Additional information: any additional information required to reanalyze the data reported in this paper is available from the lead contact upon request.

## Acknowledgments

This research was supported by the 10.13039/501100001809National Natural Science Foundation of China (project no.: 32560349), and the intelligent forest fire innovation team of higher education institutions in Guizhou Province (project no.: QJJ[2023]075). We are grateful to all the anonymous reviewers for helpful comments on earlier versions of the manuscript.

## Author contributions

Conceptualization, Y.Z.; methodology, Y.Z.; validation, Y.Z. and Z.L.; formal analysis, Y.Z., Z.L., and L.C.; resources, Y.Z.; data curation, Y.Z. and Z.L.; writing – original draft, Y.Z.; writing – review and editing, Y.Z., Z.L., and L.C.; visualization, Z.L. and L.C.; funding acquisition, Y.Z. All authors have read and agreed to the published version of the manuscript.

## Declaration of interests

The authors declare no competing interests.

## STAR★Methods

### Key resources table

**Table:** 

REAGENT or RESOURCE	SOURCE	IDENTIFIER
**Deposited data**		

Fire point data	Moderate Resolution Imaging Spectroradiometer	https://firms.modaps.eosdis.nasa.gov/download/
Forest cover data	China Multi-period Land Use Remote Sensing Monitoring Dataset	https://www.resdc.cn/DOI/DOI.aspx?DOIID=54
Meteorological factors	China Meteorological Forcing Dataset released by the National Tibetan Plateau Data Center	https://data.tpdc.ac.cn/en/data/e60dfd96-5fd8-493f-beae-e8e5d24dece4
Topographic factors	Geospatial Data Cloud Platform	https://www.geodata.cn/
Vegetation factors	National Earth System Science Data Center	https://www.geodata.cn/
Anthropogenic factors	OpenStreetMap	https://www.openstreetmap.org/directions/
Socioeconomic factors	Resource and Environmental Science Data Center	https://www.resdc.cn/

**Software and algorithms**		

R 4.4.2	Open-source software	https://cran.r-project.org/
ArcGIS 10.8	Open-source software	https://www.esri.com/en-us/hom

**Other**		

Original code for analysis	This paper	https://github.com/3078599139/code.git

### Experimental model and study participant details

Omitted as this study does not involve biological experimental models or human participants. The analysis utilized publicly available remote-sensing fire-point records and geospatial environmental datasets. Ethical approval and sex- or gender-based analyses are not applicable.

### Method details

#### Study area and overall analytical workflow

Guizhou Province is located in southwestern China (103°36′-109°35′E, 24°37′-29°13′N) (see figure below). The region has a complex topography, generally characterized by high terrain in the west and lower elevations in the east, with plateaus, mountains, and hills covering approximately 92.5% of the total area. The average annual temperature is around 15°C, with an average annual relative humidity of about 70%, and annual precipitation ranging from 1000 to 1400 mm. The region experiences a subtropical humid monsoon climate, with mild winters and warm summers. The forest resources in the study area are abundant. As of the end of 2024, the total forest area in the province was 11.14 million hectares, with a forest coverage rate of 63.3%. The vegetation types are diverse, dominated by coniferous forests such as *Pinus massoniana* and *Pinus yunnanensis*, with broadleaf forests, including *Quercus variabilis*, *Quercus glandulifera*, and *Populus* species, forming secondary communities.

**Figure:**
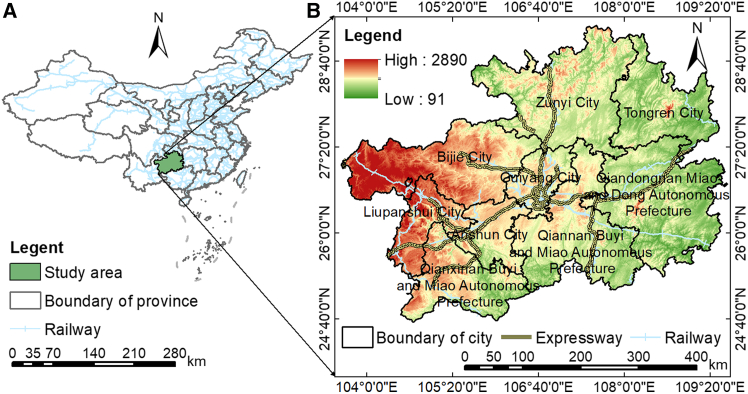
The geographical location of Guizhou Province (A) Location of Guizhou Province, China. (B) Digital elevation model (DEM) schematic diagram of Guizhou Province and partial route distribution.

The overall analytical workflow of this study consisted of five main steps: data collection, construction of fire and non-fire samples, machine-learning modeling, SHAP-based interpretation, and forest fire risk level zoning maps. The detailed workflow is shown in the figure below, and the corresponding methods are described in the method details section.

**Figure:**
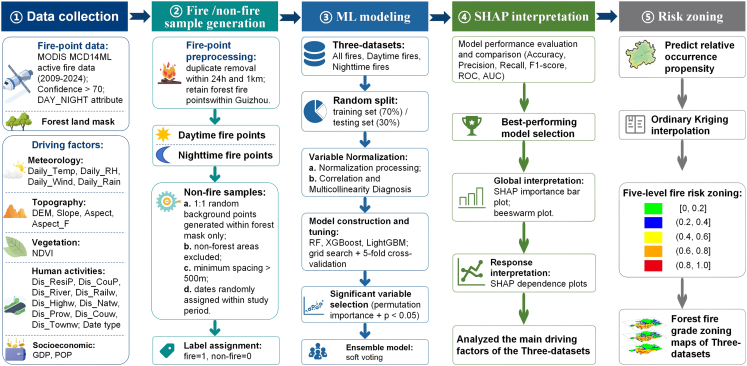
Overall workflow diagram

#### Fire point data

The fire point data used in this study were obtained from the MCD14ML dataset of the Moderate Resolution Imaging Spectroradiometer (MODIS). This dataset integrates MODIS observations from the Terra and Aqua satellites. Terra acquires data globally at approximately 10:30 and 22:30, while Aqua acquires data globally at approximately 1:30 and 13:30,^44^ enabling up to four global observations per day, significantly enhancing fire detection capabilities. It is widely used for global and regional fire dynamics monitoring and scientific research. The spatial resolution of the data is 1 km. Each fire point record contains a *Confidence* attribute field, which indicates the reliability with which a pixel is identified as an active fire point. According to the MODIS active fire product documentation, *Confidence* is a continuous numerical field ranging from 0 to 100%. To reduce the potential influence of false alarms caused by low-reliability thermal anomaly records while retaining as many valid fire point records as possible, this study used the *Confidence* field in ArcGIS 10.8 to apply an attribute filtering criterion of *Confidence* > 70. Fire point records with confidence values greater than 70% were retained for subsequent analysis. This threshold was determined with reference to the official annual forest fire statistics for Guizhou during the study period, while balancing the control of potential false detections and the retention of valid fire point samples.

On January 1, 2009, the revised *Forest Fire Prevention Regulations* were officially implemented, strengthening fire source control and legal responsibilities, marking an important turning point in the policy environment.^45^ To avoid interference from the differences in fire prevention policies during different periods and ensure the consistency of the driving factor identification, the analysis period was set after the implementation of this regulation, with the fire point data spanning from January 1, 2009, to December 31, 2024. Based on this, all fire points were classified into daytime and nighttime groups according to the DAY_NIGHT attribute field in the fire point data, enabling subsequent analysis of diurnal and nocturnal variations.

In this study, ArcGIS 10.8 was used to preprocess the obtained diurnal and nocturnal fire point data. To eliminate duplicate records caused by satellite overpasses or overlapping sensor scans from adjacent satellites, fire points within a 24-h period that were located less than 1 km apart were reduced to the earliest recorded fire point, minimizing statistical biases from repeated observations.^46^ The deduplicated fire point data were then spatially overlaid with the administrative vector boundary and forest cover data of Guizhou. Fire points located outside the province or in non-forest areas were excluded. The forest cover data were obtained from the “China Multi-period Land Use Remote Sensing Monitoring Dataset”, with a spatial resolution of 1 km. After this preprocessing, a total of 5,461 forest fire points were retained, including 4,088 daytime fire points and 1,373 nighttime fire points.

To identify and compare the main driving factors of daytime and nighttime forest fire occurrence, and to reduce the influence of class imbalance between positive and negative samples on classification model fitting and variable-importance identification, this study followed previous fire-point modeling studies and pseudo-absence sample selection methods. For the daytime and nighttime fire point datasets, non-fire points were randomly generated in ArcGIS 10.8 at a 1:1 ratio of fire points to non-fire points.^47^^,^^48^ The non-fire points were required to meet the following criteria: (1) the generation area was strictly limited to forest-covered areas in Guizhou, with water bodies, bare land, and other non-forest land-cover types excluded; (2) the spatial distance between any two points was greater than 500 m to ensure spatial independence; and (3) each non-fire point was randomly assigned a corresponding date within the study period. Subsequently, daytime and nighttime fire points were assigned a value of “1” and the corresponding non-fire points were assigned a value of “0” thus constructing binary dependent variables for the occurrence of diurnal and nocturnal forest fires.

#### Driving factor data

To systematically identify the driving mechanisms of daytime and nighttime fire occurrence, this study selected 21 factors from five categories for analysis, based on previous studies and regional conditions. Detailed information for each factor is provided in the table below.

(1) Meteorological factors: The relevant data were obtained from the China Meteorological Forcing Dataset released by the National Tibetan Plateau Data Center. The original temporal resolution of the dataset is 3 h, and the spatial resolution is 0.1°. To match the dates of fire point occurrence, temperature, relative humidity, and wind speed data within the same calendar day were averaged to obtain daily mean values, while precipitation data were summed to obtain daily cumulative precipitation, thereby generating daily-scale meteorological factors. Using the spatial analysis tools in ArcGIS 10.8, the meteorological factor values corresponding to the date and spatial location of each fire and non-fire points were extracted.
(2) Topographic factors: The base data were sourced from the 30 m resolution GDEMV2 digital elevation model released by the Geospatial Data Cloud Platform. The DEM data were processed using the spatial analysis tools in ArcGIS 10.8 to generate the corresponding slope and aspect raster layers. The raster values of each topographic factor were then extracted to the locations of all fire and non-fire points. For the aspect variable, raster values of −1 were identified as flat terrain and were separately treated as a binary variable, Aspect_F, with flat terrain assigned a value of 1 and other non-flat terrain assigned a value of 0. For non-flat pixels with raster values ranging from 0° to 360°, a continuous variable (Aspect) was used to represent the degree of slope exposure from north to south.
(3) Vegetation factors: The data were obtained from the monthly 1 km-resolution NDVI dataset for China released by the National Earth System Science Data Center. According to the month corresponding to the occurrence date of each fire point and non-fire point, the NDVI value for the corresponding month was extracted to characterize the vegetation cover and fuel background conditions during the period of fire point occurrence. Using the spatial analysis tools in ArcGIS 10.8, the NDVI data corresponding to the month of each fire and non-fire points were extracted to their specific geographic locations.
(4) Anthropogenic factors: Human activity impacts were quantified from the perspectives of forest accessibility and temporal activity patterns. Spatially, the euclidean distance to residential points, county points, rivers, railways, expressways, national highway, provincial highways, county highway, and township roads were selected as indicators of forest accessibility. All base vector data were obtained from OpenStreetMap. Using ArcGIS 10.8 proximity analysis tools, the shortest straight-line distance from each fire and non-fire points to the above-mentioned features was calculated. Temporally, to analyze the impact of periodic human activities on fire risk, the date type was introduced as a categorical variable, dividing all dates into three categories: Ⅰ: weekdays, Ⅱ: public holidays, Ⅲ: weekends. When weekends and public holidays overlapped, they were prioritized as public holidays. The corresponding date type was assigned to each fire and non-fire point based on their respective dates. These factors were spatially relatively stable and were therefore not temporally matched.
(5) Socioeconomic factors: Data were sourced from the Resource and Environmental Science Data Center with a 1 km-resolution raster dataset. This dataset includes GDP and population density (POP) for four reference years: 2005, 2010, 2015 and 2019. To obtain complete annual data for the study period, the raster calculator in ArcGIS 10.8 was used to generate GDP and POP raster data for the remaining years based on the average rate of change between adjacent benchmark years. The spatial analysis tools were then used to extract the corresponding annual GDP and POP data according to the year associated with each fire and non-fire points.

**Table:** Selected independent variables for forest fires in the study

Feature	Variables	Abbreviation	Unit	Numerical range	Variable type
Meteorological	daily average temperature	Daily_Temp	°C	1–32.4788	numerical
	daily average relative humidity	Daily_RH	%	13.0913-98.3737	numerical
	daily average wind speed	Daily_Wind	m/s	0.1300-9.7362	numerical
	daily accumulated precipitation	Daily_Rain	mm	0–30.6720	numerical
Topographic	digital elevation model	Dem	m	257–2582	numerical
	slope	Slope	°	0–69.0819	numerical
	aspect (flat)	Aspect_F		−1	categorical
	aspect	Aspect	°	0–359.6210	numerical
Vegetation	normalized difference vegetation index	NDVI		0–0.8786	numerical
Anthropogenic	distance from residential points	Dis_ResiP	km	0.0190-5.2687	numerical
	distance from county points	Dis_CouP		0.3510-52.3631	numerical
	distance from river	Dis_River		0.0044-40.4890	numerical
	distance from railway	Dis_Railw		0.0119-98.9930	numerical
	distance from expressways	Dis_Highw		0.0030–122.0420	numerical
	distance from national highway	Dis_Natw		0.0027–99.0911	numerical
	distance from provincial highway	Dis_Prow		0.0011–43.5710	numerical
	distance from county highway	Dis_Couw		0.0020-17.8300	numerical
	distance from township road	Dis_Townw		0.0141-28.9071	numerical
	date type	Date_T	day	Ⅰ-Ⅲ	categorical
Socioeconomic	gross domestic product	GDP	10,000 yuan	0–8961	numerical
	population density	POP	number/km^2^	31.2–9107	numerical

Subsequent variable expressions will be represented by the abbreviations shown in the table.

The temporal matching scale of each driving factor in this study was determined according to its ecological significance and data availability. The daily-scale meteorological factors used mainly reflect the overall meteorological background on the day of fire point occurrence and cannot fully capture the instantaneous meteorological differences between daytime and nighttime satellite overpass times. Therefore, the model results are mainly used to explain the driving differences between daytime and nighttime forest fire points under daily-scale environmental backgrounds, rather than the hourly scale fire triggering process.

#### Variable normalization processing

To eliminate the influence of differences in the dimensions and value ranges of explanatory variables on model training, and to improve model convergence speed and stability,^49^ all continuous explanatory variables were normalized in this study. Slope was transformed using a sine function, as shown in Equation 1. For the continuous aspect variable, the original aspect values were transformed using a south-facing index, as shown in Equation 2. The other continuous variables were processed using the minimum–maximum normalization method, by which the original data were linearly transformed into the range of [0, 1], as shown in Equation 3.

(Equation 1) $$S^{*}=\sin\frac{\pi S}{180}$$

(Equation 2) $$A_{s}=\frac{1-\cos\left(\frac{\pi \theta }{180}\right)}{2}$$

(Equation 3) $$X^{*}=\frac{X_{j}-X_{j,\min}}{X_{j,\max}-X_{j,\min}}$$

where *S∗*, *A*_*s*_, and *X∗* denote the normalized values; *S* represents the original Slope value; *θ* represents the original Aspect angle; and *X*_*j,min*_ and *X*_*j,max*_ represent the minimum and maximum values of the *j* th continuous environmental factor, respectively.

#### Data preprocessing and exploratory analysis

##### Spatiotemporal pattern analysis of fire points

To reveal the temporal variations and spatial distribution characteristics of forest fire points in Guizhou from 2009 to 2024, this study conducted analyses from both temporal and spatial dimensions. In the temporal dimension, the cumulative numbers of all, daytime, and nighttime forest fire points were calculated at annual, monthly, and satellite overpass detection-hour scales, respectively, and corresponding bar charts were produced to characterize the long-term variation trend, seasonal distribution pattern, and day–night activity differences of forest fire points. In the spatial dimension, based on the geographic coordinates of daytime and nighttime forest fire points, kernel density analysis in ArcGIS 10.8 was used to generate spatial density distribution maps, which were used to identify high-density aggregation areas of forest fire points under different temporal scenarios and their spatial differences.

##### Data partitioning

To analyze differences in the drivers of forest fire point occurrence under different temporal scenarios, three analytical datasets were constructed in this study: all, daytime, and nighttime fire points. Each dataset included forest fire points, corresponding non-fire samples, and their matched driving factors. For the three datasets, stratified random sampling was performed separately in R 4.4.2, using the binary classification label of fire occurrence or non-occurrence as the stratification criterion.^50^ The data were divided into training and testing sets at a ratio of 7:3. This method ensured that the proportions of fire and non-fire samples in the training and testing sets were consistent with those in the corresponding original dataset, thereby reducing class-distribution bias caused by random partitioning. To prevent information from the testing set from being involved in variable screening and model construction, the testing set was used only to evaluate the predictive performance of the final models, and all subsequent analyses were conducted based on the training set.

##### Correlation and multicollinearity diagnosis

To evaluate the degree of information overlap among candidate driving factors and its potential influence on model interpretation, correlation and multicollinearity diagnoses were conducted separately based on the training sets of the all, daytime, and nighttime datasets. Spearman rank correlation analysis was first performed for continuous candidate driving factors. When the absolute value of the correlation coefficient between two variables was |r_s_| ≥ 0.70, the variables were considered to have a strong correlation.^51^^,^^52^ Subsequently, multicollinearity diagnosis was further conducted for the candidate variables. The variance inflation factor (VIF) was used to evaluate continuous variables and the binary variable Aspect_F, whereas the generalized variance inflation factor (GVIF) was used to evaluate the multi-category variable Date_T. Comparisons were made using the corrected equivalent value, (GVIF^1/(2Df)^).^2^ When the VIF or the equivalent value (GVIF^1/(2Df)^)^2^ was less than 10, no severe multicollinearity was considered to exist among the candidate variables.^53^ The above diagnoses were mainly used to evaluate the reliability of variable-importance rankings and SHAP interpretation results, rather than as a prerequisite for excluding variables with clear ecological meaning.

#### Construction and optimization of basic models

The optimization of parameters, variable selection, and model validation were all implemented in R 4.4.2.

##### Model principle

RF model is an ensemble model based on the Bagging approach.^54^ It generates multiple training subsets from the original training set through bootstrap sampling and constructs decision trees independently for each subset. During the training of each decision tree, a random subset of features is selected for node splitting. All decision trees are grown independently until they are fully matured, introducing randomness and enhancing model diversity. Finally, the model’s prediction is made by aggregating the results from all decision trees through a voting mechanism. This method not only offers strong robustness and training efficiency but also demonstrates good tolerance to high-dimensional data, effectively reducing the overfitting risk of individual decision trees.^55^

XGBoost model is an optimized algorithm based on the gradient boosting decision tree method.^56^ It excels in classification and regression tasks by incorporating various improvements and is widely used in fields such as data mining and computer vision.^57^ The core idea of XGBoost is to begin with a simple weak learner and sequentially train new decision trees, where each new tree focuses on fitting the residuals of the predictions from the previous ensemble model, progressively correcting errors to enhance model performance. Its objective function consists of two parts: the training loss term, which measures the deviation between predicted and actual values, and the regularization term (including L1 and L2 regularization), which constrains the complexity of the decision trees and effectively prevents overfitting.

LightGBM model is an efficient machine learning algorithm based on the gradient boosting framework, particularly suitable for large-scale datasets.^58^ Its key innovation lies in the use of a histogram-based splitting strategy that discretizes continuous features into a fixed number of bins, significantly reducing computation and memory usage. The algorithm adopts a leaf-wise growth strategy with depth constraints, replacing the traditional level-wise splitting strategy, further improving training speed and efficiency.

##### Hyperparameter optimization

To fully leverage the performance of each model and avoid overfitting while improving generalization, we performed hyperparameter optimization for the three models. The optimization process was based on each type of training dataset, and a combination of grid search and 5-fold cross-validation was used. Using the average performance of cross-validation as the evaluation criterion, the optimal parameter combination was obtained (Table S5). The obtained optimal parameters were used for subsequent model training, significant predictor screening, and ensemble model construction.

The RF model included two core parameters: *mtry*, which controls the number of features randomly sampled for each decision tree, and *ntree*, which controls the total number of decision trees. The out-of-bag error rate was used as the optimization criterion. The search range for *mtry* was set to [2, 10] with a step size of 1, and the search range for *ntree* was set to (0, 1000] with a step size of 100. This model was built and tuned via the *randomForest* package, and its hyperparameter optimization is depicted in Figure S1.

The XGBoost model included three core parameters: *eta*, which controls the contribution weight of each tree to the final model and helps prevent overfitting; *max_depth*, which controls model complexity; and *colsample_bytree*, which controls the proportion of features sampled for each tree to increase randomness. In this study, Log loss was used as the optimization criterion. The search range for *eta* was set to [0.01, 0.1, 0.15, 0.2], the search range for *max_depth* was set to [4, 5, 6, 7], and the search range for *colsample_bytree* was set to [0.4, 0.6, 0.8, 1.0]. Using the *xgboost* package, we conducted both parameter tuning and model construction; the hyperparameter optimization procedure is presented in Figure S2.

The LightGBM model included three core parameters: *learning_rate*, which has the same function as *eta* in XGBoost; *num_leaves*, which is the main parameter controlling model complexity; and *feature_fraction*, which has a function similar to that of *colsample_bytree*. In this study, log loss was used as the optimization criterion. The search range for *learning_rate* was set to [0.01, 0.05, 0.1, 0.15, 0.2], the search range for *num_leaves* was set to [31, 63, 127, 255], increasing according to 2^*n*^-1, where *n* = 5, 6, 7, and 8, and the search range for *feature_fraction* was set to [0.4, 0.6, 0.8, 1.0]. This model was built and tuned via the *lightgbm* package, and its hyperparameter optimization is depicted in Figure S3.

##### Selection of significant independent variables

To further identify the driving factors that had a significant influence on forest fire occurrence, this study used the permutation importance method to screen variables for the parameter-tuned RF, XGBoost, and LightGBM models separately. This method evaluates feature importance by randomly permuting the values of a given feature and observing the resulting decrease in model performance, measured by the AUC value. The results are generally stable and interpretable. For each model, 100 permutation experiments were conducted sequentially for each independent variable based on the corresponding training set. The decrease in model AUC after each permutation was calculated, and the mean decrease was used as the importance score of that variable. Meanwhile, a one-sided test was performed based on the permutation distribution to calculate the *p* value. Variables with *p* < 0.05 were identified as factors with a significant influence on forest fire occurrence in the corresponding model. This analysis was conducted independently for the daytime, nighttime, and all-fire datasets. The significant factors shared by the three models were extracted and defined as the final main driving factors, thereby obtaining the significant variables corresponding to each model under different temporal scenarios.

#### Ensemble model and comprehensive evaluation

##### Construction of ensemble model

After completing the hyperparameter optimization and significant variable selection for each model, we used the soft voting method to construct an ensemble model in order to integrate the predictive advantages of different algorithms. First, using the selected key driving factors, the three models were retrained on the training set to ensure that each model was built with the optimal influencing variables. Subsequently, the fire occurrence probabilities from the outputs of the three models were obtained on the testing set, and their arithmetic mean was calculated as the final predicted probability for the ensemble model. If this average probability was greater than 0.5, the fire occurrence was predicted; otherwise, it was predicted not to occur. This method not only combines the discriminative information from each model but also retains the uncertainty measurement of the predictions, which helps to improve the overall robustness and prediction accuracy of the model. The Ensemble Model will be used for subsequent comprehensive performance evaluation and comparative analysis.

##### Model performance evaluation and comparison

To comprehensively evaluate the predictive performance of the RF, XGBoost, LightGBM, and Ensemble Model, a comparative analysis was conducted using the testing set. The performance evaluation metrics include Accuracy, Precision, Recall, F1-score, and the Area Under the Receiver Operating Characteristic Curve (ROC, AUC).^59^ The ROC curve is plotted with Sensitivity on the y axis and 1-Specificity on the x axis, with each point reflecting the model’s performance at different thresholds. AUC, as an overall accuracy measure, indicates better classification performance with higher values. The relevant formulas for these metrics are provided in Equations 4, 5, 6 and 7.

(Equation 4) $$A\text{ccuracy}=\frac{TP+TN}{TP+FP+TN+FN}$$

(Equation 5) $$P\text{recision}=\frac{\text{TP}}{\text{TP}+\text{FP}}$$

(Equation 6) $$R\text{ecall}=\frac{TP}{TP+FN}$$

(Equation 7) $$F1=2\times \frac{P\text{recision}\times R\text{ecall}}{P\text{recision}+R\text{ecall}}$$

In binary classification evaluation, *TP* represents the number of samples correctly predicted by the model as forest fire occurrences; *FP* represents the number of samples incorrectly predicted by the model as forest fire occurrences when they were actually non-fire samples; *FN* represents the number of samples incorrectly predicted by the model as non-fire occurrences when they were actually forest fire samples; and *TN* represents the number of samples correctly predicted by the model as non-fire occurrences.

##### Model interpretation based on SHAP

Based on the best-performing model, SHapley Additive exPlanations (SHAP) were used to conduct an interpretability analysis of the selected significant influencing factors, further explaining the prediction mechanisms and feature contributions of the variables. SHAP analysis not only quantifies the importance of each variable from a statistical perspective but also reveals the model’s decision-making logic from a mechanistic standpoint.^60^ By calculating the Shapley value of each variable for the model’s prediction, SHAP fairly attributes complex nonlinear predictions to individual input variables, providing consistent and quantifiable explanations at both global and local levels. At the global level, we used SHAP feature importance bar charts to display the magnitude of the average absolute SHAP values of each feature, identifying the factors with the most significant impact on forest fire occurrence risk. Meanwhile, the SHAP beeswarm plot further reveals the distribution relationship between the actual values of features and their contribution directions, visually presenting the heterogeneity of feature effects across different samples and their association with prediction outcomes. At the local level, SHAP dependency plots were created for the main driving factors. These plots reflect the nonlinear trajectory of a single feature’s value changes and their contribution to model predictions, helping to explain how each factor specifically influences the probability of forest fire occurrence under certain environmental conditions.

#### Forest fire grade zoning maps

Based on the optimal model, prediction values for forest fire points were obtained for three-datasets in Guizhou. The model-predicted values were used to represent the relative occurrence propensity of forest fire points, rather than absolute occurrence probabilities. The ordinary kriging interpolation method in ArcGIS 10.8 was used to transform the predicted values at sample points into spatially continuous surfaces to display their spatial differentiation characteristics. According to the prediction value range of [0, 1], an equal-interval classification method was used to divide the values into five grades: Grade I, extremely low-risk zone [0, 0.2]; Grade II, low-risk zone (0.2, 0.4]; Grade III, moderate-risk zone (0.4, 0.6]; Grade IV, high-risk zone (0.6, 0.8]; and Grade V, extremely high-risk zone (0.8, 1.0]. The generated grade zoning maps were used to identify relatively susceptible areas of forest fire points in Guizhou and their spatial differences between daytime and nighttime scenarios, providing a spatial reference for regional forest fire patrols, ignition-source control, and differentiated fire prevention and management.

### Quantification and statistical analysis

All statistical analyses and machine-learning procedures were performed in R 4.4.2, and spatial analyses were conducted in ArcGIS 10.8. Fire and non-fire samples were coded as 1 and 0, respectively. For the all-fire, daytime, and nighttime datasets, stratified random sampling was used to split the data into training and testing sets at a ratio of 7:3, with the testing set used only for final model evaluation. Spearman’s rank correlation analysis was used to assess correlations among continuous predictors, with |rs| ≥ 0.70 indicating strong correlation. Multicollinearity was evaluated using VIF and GVIF, with corrected (GVIF^1/(2Df)^)^2^ values used for categorical variables; values < 10 were considered to indicate no severe multicollinearity. Model performance was evaluated using accuracy, precision, recall, F1-score, ROC curves, and AUC. Significant predictors were identified by permutation importance based on 100 permutations of AUC decrease, and statistical significance was assessed using a one-sided permutation test, with *p* < 0.05 considered statistically significant. SHAP analysis was used to interpret predictor contributions, with mean absolute SHAP values representing overall variable importance.
