# Supplementary material for: Interpretable machine learning reveals daytime and nighttime forest fire point drivers in Guizhou
Source: iScience. 2026 Jul 15;29(8):116799. doi: 10.1016/j.isci.2026.116799 (PMC13383953; doi:10.1016/j.isci.2026.116799)
Supplement: Document S1. Figures S1–S3 and Tables S1–S5 [file mmc1.pdf]

iScience, Volume *29*

Supplemental information

Interpretable machine learning reveals daytime and nighttime forest fire point drivers in Guizhou

Yunlin Zhang, Zhiyang Li, and Long Chen

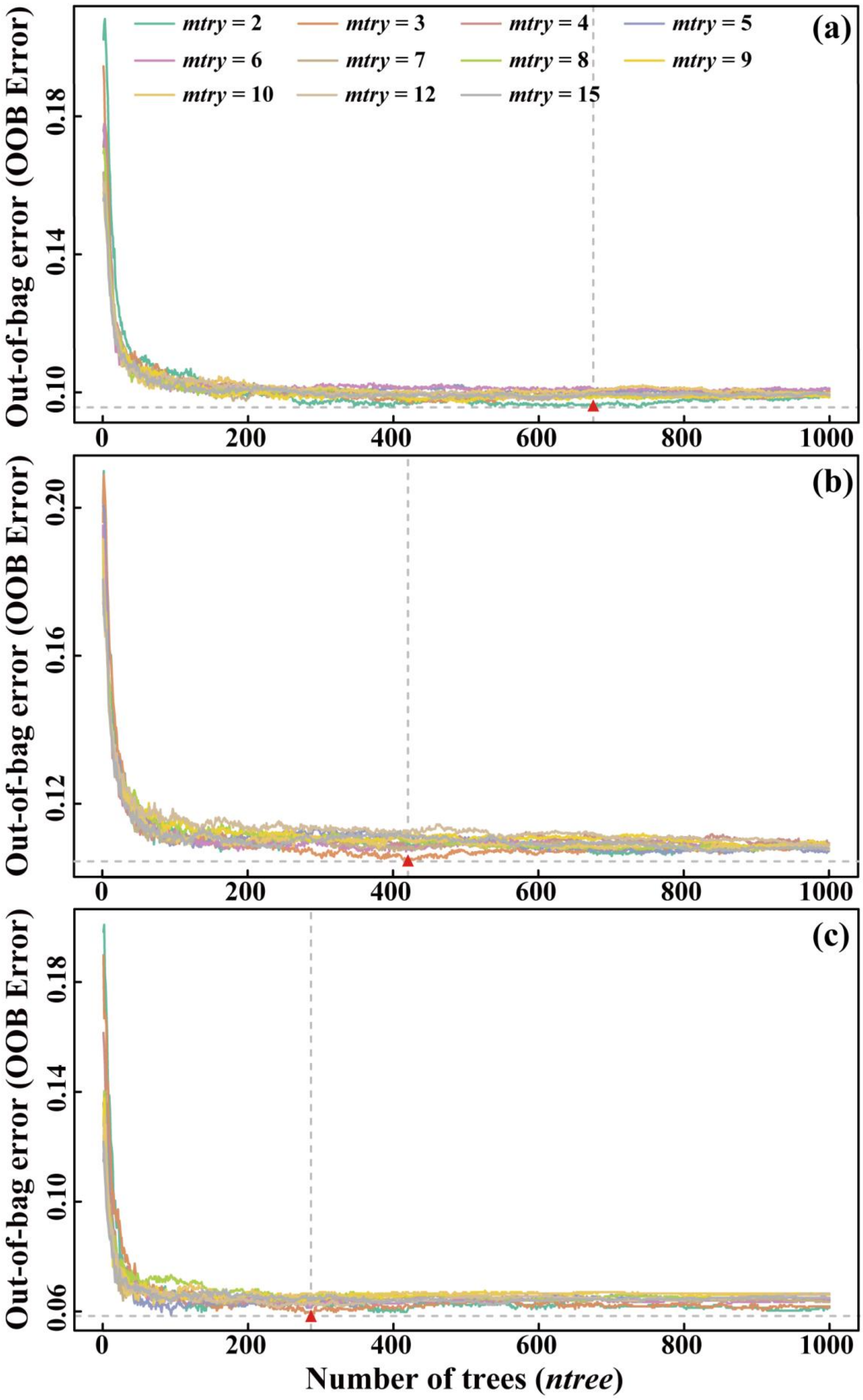

Figure S1. Hyperparameter tuning results for the RF model, related to STAR Methods. The position of the red triangle indicates the optimal combination of model parameters.

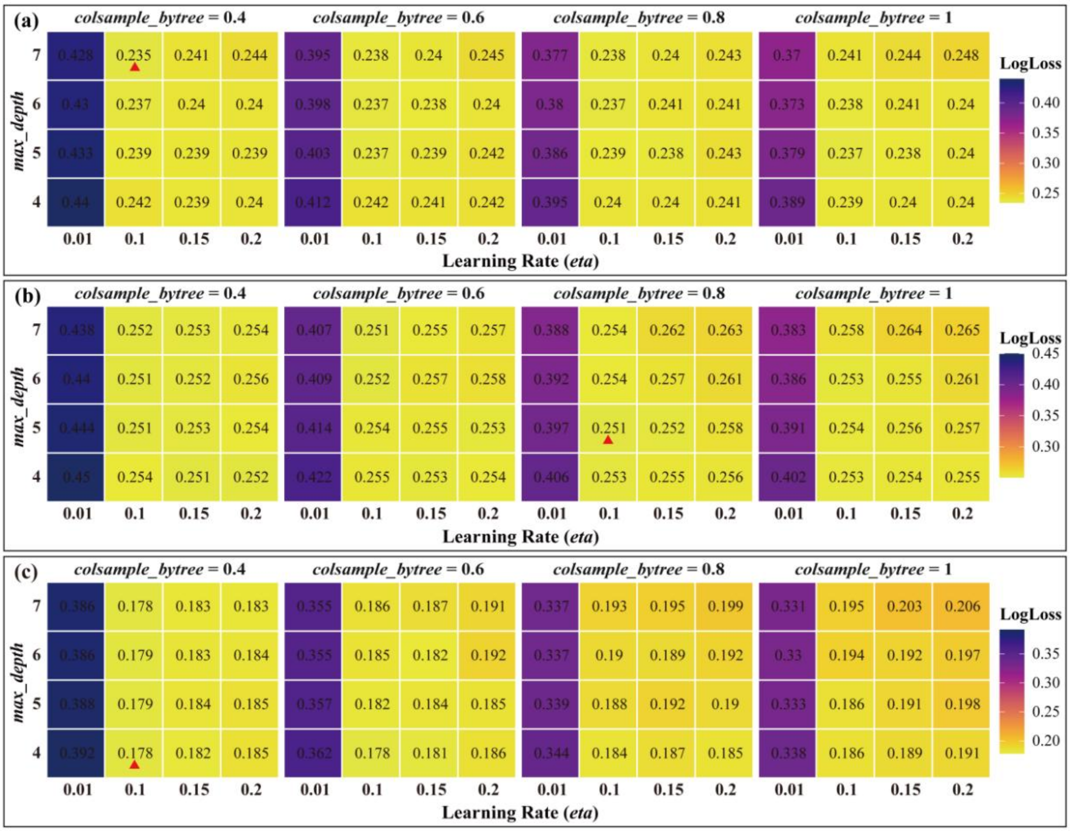

Figure S2. Hyperparameter tuning results for the XGBoost model, related to STAR Methods. The position of the red triangle indicates the optimal combination of model parameters.

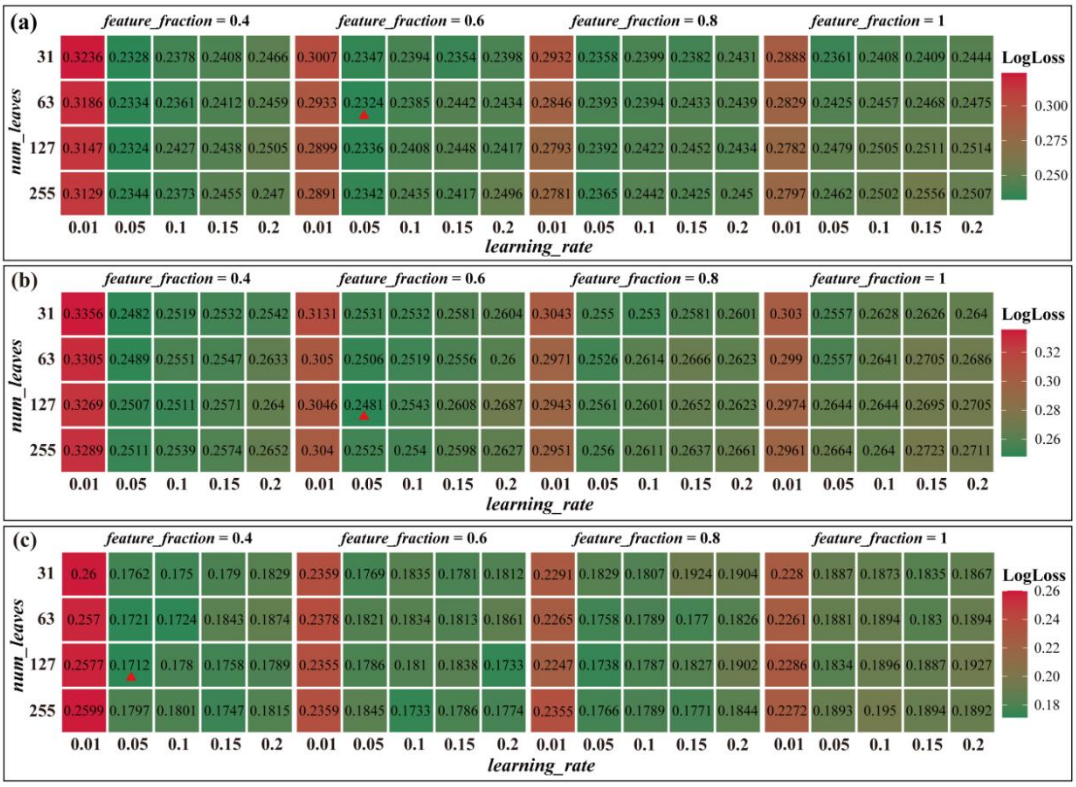

Figure S3. Hyperparameter tuning results for the LightGBM model, related to STAR Methods. The position of the red triangle indicates the optimal combination of model parameters.

Table S1. Selection of significant variables using three datasets and three models, related to STAR Methods. "＋" indicates that the algorithm selected the variable as the primary driver. "－" indicates that the algorithm did not select the variable as the primary driver.

Variable	Full				Daytime				Nighttime			
	**RF**	**XGBoost**	**LightGBM**	**Mutual**	**RF**	**XGBoost**	**LightGBM**	**Mutual**	**RF**	**XGBoost**	**LightGBM**	**Mutual**
Daily_RH	＋	＋	＋	＋	＋	＋	＋	＋	＋	＋	＋	＋
NDVI	＋	＋	＋	＋	＋	＋	＋	＋	＋	＋	＋	＋
Daily_Rain	＋	＋	＋	＋	＋	＋	＋	＋	＋	＋	＋	＋
Daily_Wind	＋	＋	＋	＋	＋	＋	＋	＋	＋	＋	＋	＋
Daily_Temp	＋	＋	＋	＋	＋	＋	＋	＋	＋	＋	＋	＋
Dis_ResiP	＋	＋	＋	＋	＋	＋	＋	＋	＋	＋	＋	＋
Dis_Highw	＋	＋	＋	＋	＋	＋	＋	＋	＋	＋	＋	＋
GDP	＋	＋	＋	＋	＋	＋	＋	＋	＋	＋	－	－
Dem	＋	＋	＋	＋	＋	＋	＋	＋	＋	＋	－	－
Dis_Natw	＋	＋	＋	＋	＋	＋	＋	＋	＋	＋	－	－
Dis_River	＋	＋	－	－	＋	＋	－	－	－	－	－	－
POP	＋	＋	－	－	＋	＋	＋	＋	－	＋	－	－
Dis_Coup	＋	＋	－	－	＋	－	－	－	－	－	－	－
Dis_Prow	＋	＋	－	－	＋	＋	＋	＋	－	＋	－	－
Dis_Railw	＋	＋	－	－	＋	＋	－	－	＋	＋	＋	＋
Slope	－	＋	－	－	＋	－	＋	－	＋	＋	－	－
Dis_Couw	－	－	－	－	＋	－	－	－	＋	＋	－	－
Aspect	－	＋	－	－	＋	－	－	－	－	＋	－	－
Dis_Townw	－	－	－	－	－	－	－	－	－	＋	－	－
Date_T	－	－	－	－	－	－	－	－	－	＋	－	－
Aspect_F	－	－	－	－	－	－	－	－	－	－	－	－

Table S2. Permutation importance results of the independent variables for the full dataset, related to STAR Methods. Predictor significance was assessed using a one-sided permutation test based on 100 permutations of AUC decrease. Bolded values represent predictors with $p < 0.05$, which were considered statistically significant.

ID	Variable	RF		XGBoost		LightGBM	
		Importance	p_value	Importance	p_value	Importance	p_value
1	Daily_RH	0.03769	**0.00**	0.09509	**0.00**	0.10298	**0.00**
2	NDVI	0.01089	**0.00**	0.03917	**0.00**	0.03812	**0.00**
3	Daily_Rain	0.00144	**0.00**	0.00423	**0.00**	0.00455	**0.00**
4	Daily_Wind	0.00130	**0.00**	0.00417	**0.00**	0.00388	**0.00**
5	Daily_Temp	0.00084	**0.00**	0.00833	**0.00**	0.00996	**0.00**
6	Dis_ResiP	0.00079	**0.00**	0.00326	**0.00**	0.00320	**0.00**
7	Dis_Highw	0.00010	**0.00**	0.00222	**0.00**	0.00182	**0.00**
8	GDP	0.00008	**0.00**	0.00215	**0.00**	0.00130	**0.00**
9	Dem	0.00007	**0.00**	0.00212	**0.00**	0.00139	**0.00**
10	Dis_Natw	0.00007	**0.00**	0.00165	**0.00**	0.00135	**0.00**
11	Dis_River	0.00003	**0.00**	0.00097	**0.00**	0.00048	1.00
12	POP	0.00001	**0.00**	0.00157	**0.00**	0.00083	0.06
13	Dis_Coup	0.00001	**0.00**	0.00096	**0.00**	0.00062	1.00
14	Dis_Prow	0.00001	**0.00**	0.00089	**0.00**	0.00066	0.06
15	Dis_Railw	0.00001	**0.00**	0.00083	**0.00**	0.00057	1.00
16	Slope	0.00001	0.06	0.00088	**0.00**	0.00039	1.00
17	Dis_Couw	0.00000	0.06	0.00057	1.00	0.00046	1.00
18	Aspect	0.00000	0.07	0.00075	**0.00**	0.00035	1.00
19	Dis_Townw	0.00000	0.07	0.00065	1.00	0.00036	1.00
20	Date_T	0.00000	0.10	0.00037	1.00	0.00019	1.00
21	Aspect_F	0.00000	1.00	0.00000	1.00	0.00000	1.00

Table S3. Permutation importance results of the independent variables for the daytime dataset, related to STAR Methods. Predictor significance was assessed using a one-sided permutation test based on 100 permutations of AUC decrease. Bolded values represent predictors with $p < 0.05$, which were considered statistically significant.

ID	Variable	RF		XGBoost		LightGBM	
		Importance	p_value	Importance	p_value	Importance	p_value
1	Daily_RH	0.05822	**0.00**	0.11286	**0.00**	0.05445	**0.00**
2	NDVI	0.02426	**0.00**	0.06191	**0.00**	0.02509	**0.00**
3	Daily_Rain	0.00745	**0.00**	0.00897	**0.00**	0.00299	**0.00**
4	Daily_Wind	0.00679	**0.00**	0.00732	**0.00**	0.00292	**0.00**
5	Daily_Temp	0.00559	**0.00**	0.01818	**0.00**	0.00550	**0.00**
6	Dis_ResiP	0.00447	**0.00**	0.00687	**0.00**	0.00156	**0.00**
7	Dis_Highw	0.00269	**0.00**	0.00352	**0.00**	0.00015	**0.00**
8	GDP	0.00238	**0.00**	0.00386	**0.00**	0.00029	**0.00**
9	Dem	0.00224	**0.00**	0.00372	**0.00**	0.00013	**0.00**
10	Dis_Natw	0.00233	**0.00**	0.00276	**0.00**	0.00012	**0.00**
11	Dis_River	0.00151	**0.00**	0.00176	**0.00**	0.00000	0.19
12	POP	0.00140	**0.00**	0.00267	**0.00**	0.00007	**0.00**
13	Dis_Coup	0.00119	**0.00**	0.00138	0.68	0.00000	0.14
14	Dis_Prow	0.00117	**0.00**	0.00172	**0.00**	0.00006	**0.00**
15	Dis_Railw	0.00141	**0.00**	0.00167	**0.00**	0.00005	0.06
16	Slope	0.00116	**0.00**	0.00152	0.65	0.00000	**0.00**
17	Dis_Couw	0.00117	**0.00**	0.00073	1.00	0.00000	0.08
18	Aspect	0.00112	**0.00**	0.00137	0.90	0.00000	0.07
19	Dis_Townw	0.00105	0.98	0.00074	1.00	0.00000	0.36
20	Date_T	0.00091	1.00	0.00095	0.90	0.00000	0.11
21	Aspect_F	0.00000	1.00	0.00000	1.00	0.00000	1.00

Table S4. Permutation importance results of the independent variables for the nighttime dataset, related to STAR Methods. Predictor significance was assessed using a one-sided permutation test based on 100 permutations of AUC decrease. Bolded values represent predictors with $p < 0.05$, which were considered statistically significant.

ID	Variable	RF		XGBoost		LightGBM	
		Importance	p_value	Importance	p_value	Importance	p_value
1	Daily_RH	0.08731	**0.00**	0.14564	**0.00**	0.04675	**0.00**
2	NDVI	0.00449	**0.00**	0.01593	**0.00**	0.00116	**0.00**
3	Daily_Rain	0.00025	**0.00**	0.00079	**0.00**	0.00004	**0.00**
4	Daily_Wind	0.00038	**0.00**	0.00175	**0.00**	0.00017	**0.00**
5	Daily_Temp	0.00063	**0.00**	0.00411	**0.00**	0.00027	**0.00**
6	Dis_ResiP	0.00027	**0.00**	0.00186	**0.00**	0.00005	**0.00**
7	Dis_Highw	0.00004	**0.01**	0.00125	**0.00**	0.00005	**0.00**
8	GDP	0.00002	**0.01**	0.00132	**0.00**	0.00000	0.73
9	Dem	0.00001	**0.01**	0.00073	**0.00**	0.00000	0.90
10	Dis_Natw	0.00011	**0.00**	0.00099	**0.00**	0.00002	0.08
11	Dis_River	0.00000	0.14	0.00023	0.09	0.00000	1.00
12	POP	0.00000	0.10	0.00060	**0.00**	0.00000	1.00
13	Dis_Coup	0.00000	0.58	0.00016	0.10	0.00000	1.00
14	Dis_Prow	0.00000	0.39	0.00044	**0.00**	0.00000	1.00
15	Dis_Railw	0.00000	**0.03**	0.00028	**0.00**	0.00003	**0.00**
16	Slope	0.00001	**0.01**	0.00048	**0.00**	0.00000	1.00
17	Dis_Couw	0.00000	**0.04**	0.00055	**0.00**	0.00000	1.00
18	Aspect	0.00000	0.98	0.00047	**0.00**	0.00000	1.00
19	Dis_Townw	0.00000	0.31	0.00035	**0.00**	0.00000	1.00
20	Date_T	0.00000	0.56	0.00003	**0.01**	0.00000	1.00
21	Aspect_F	0.00000	1.00	0.00000	1.00	0.00000	1.00

Table S5. Optimized hyperparameter combinations of the three machine learning models, related to STAR Methods. Hyperparameters not listed in the table were retained at their default settings. RF, Random Forest; XGBoost, eXtreme Gradient Boosting; LightGBM, Light Gradient Boosting Machine.

Dataset	Model	Optimized hyperparameter
Full	RF	mtry = 2, ntree = 675
	XGBoost	eta = 0.1, max_depth = 7, colsample_bytree = 0.4
	LightGBM	learning_rate = 0.05, num_leaves = 63, feature_fraction = 0.6
Daytime	RF	mtry = 3, ntree = 420
	XGBoost	eta = 0.1, max_depth = 5, colsample_bytree = 0.8
	LightGBM	learning_rate = 0.05, num_leaves = 127, feature_fraction = 0.6
Nighttime	RF	mtry = 3, ntree = 282
	XGBoost	eta = 0.1, max_depth = 4, colsample_bytree = 0.4
	LightGBM	learning_rate = 0.05, num_leaves = 127, feature_fraction = 0.4
